# Unilateral and Spontaneous Complete Anterior Dislocation of the Crystalline Lens in a Patient With Homocystinuria

**DOI:** 10.7759/cureus.14655

**Published:** 2021-04-23

**Authors:** Arezoo Miraftabi, Amin Zand, Kaveh Abri Aghdam

**Affiliations:** 1 Department of Ophthalmology, Eye Research Center, The Five Senses Institute, Rassoul Akram Hospital, Iran University of Medical Sciences, Tehran, IRN

**Keywords:** homocystinuria, crystalline lens, dislocation, subluxation

## Abstract

Homocystinuria is a metabolic disorder caused by a deficiency of cystathionine beta-synthase with autosomal recessive inheritance. Clinically it is characterized by lens subluxation, skeletal abnormalities, and thromboembolic accidents. We present a 6-year-old boy who was a known case of homocystinuria. The patient had a previous history of thrombotic cerebrovascular infarction at the age of 3. He had mild and vague pain in the left eye two weeks before presentation without being exposed to trauma. Ophthalmic examination revealed the dislocation of the crystalline lens into the anterior chamber with diffuse corneal stromal edema in the affected eye. The patient was treated with topical atropine and betamethasone eye drops, but due to the corneo-lenticular contact and corneal edema, he underwent lens extraction and placement of iris-fixated intraocular lens after 48 hours. Corneal edema exhibited improvement at follow-up visits. Early age onset and unilateral complete lens dislocation to the anterior chamber in the absence of a history of trauma is a less common presentation of homocystinuria. In patients with systemic diseases including homocystinuria that cause zonulysis, lens dislocation is usually symmetric and bilateral. Nevertheless, in unilateral cases especially in those who did not have any history of trauma, evaluation for systemic diseases like homocystinuria is necessary for early diagnosis and prevention from other systemic involvements.

## Introduction

Ectopia lentis (subluxation or dislocation of the crystalline lens) can occur due to trauma or some certain systemic diseases including homocystinuria [1]. Homocystinuria is an autosomal recessive metabolic disease caused by the absence of cystathionine β-synthase. It is associated with osteoporosis, thrombotic events, and mental disorders [2]. Ectopia lentis in homocystinuria is usually bilateral and symmetric with inferonasal displacement due to decreased zonular integrity [3]. We report a young child with unilateral complete anterior lens dislocation and subsequent corneal edema without any previous history of trauma, as a less common ocular presentation of homocystinuria. The following case is presented in accordance with the CARE guidelines for case reports (available at https://www.care-statement.org/checklist).

## Case presentation

**Figure 1:**
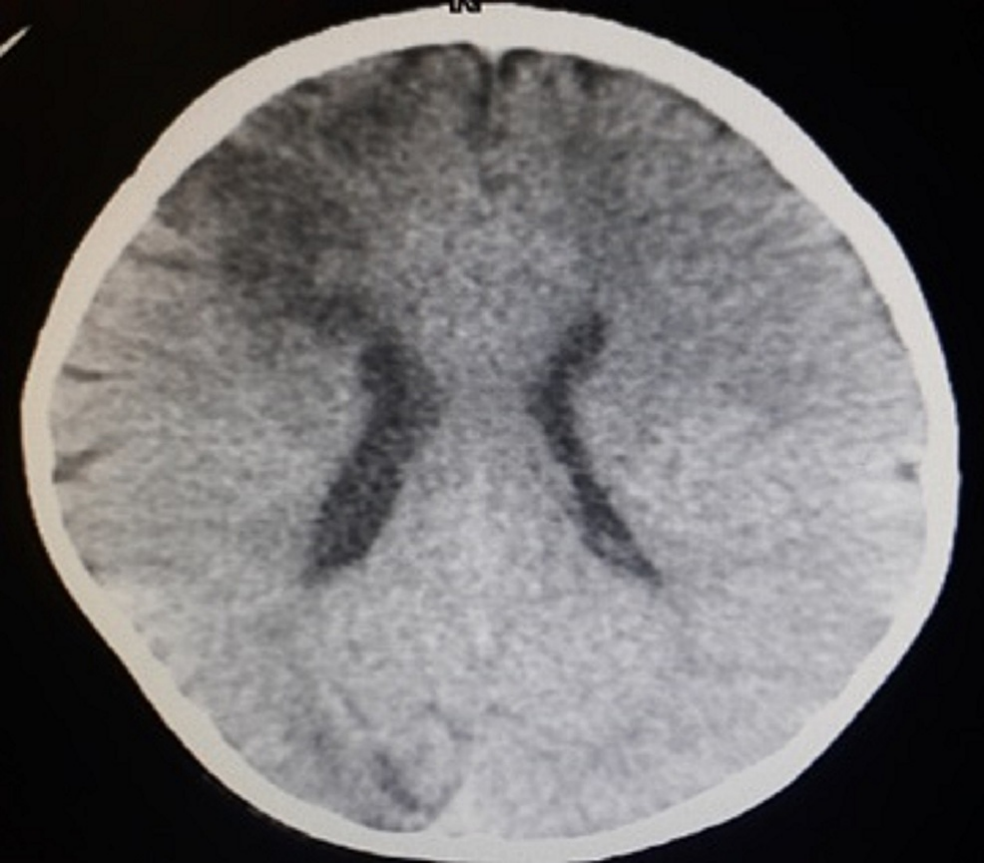
Non-contrast axial brain computed tomography (CT) image showing a hypodense area in the right hemisphere due to an old infarction.

The patient was a 6-year-old boy with some cognitive disability. He was a known case of homocystinuria. He complained of mild and vague ocular pain of the left eye two weeks before presentation to the clinic. Due to progressive corneal haziness in recent days, noticed by his parents, he was brought to our clinic. His medical history was significant for a cerebrovascular accident with acquired cerebral palsy 1.5 years ago. It presented as a hypodense lesion in the right hemisphere of the brain in a computed tomography (CT) scan (Figure 1).

The family history was unremarkable and his parents were not relatives. There was no history of trauma. He refused to wear his high-myopic glasses.

**Figure 2:**
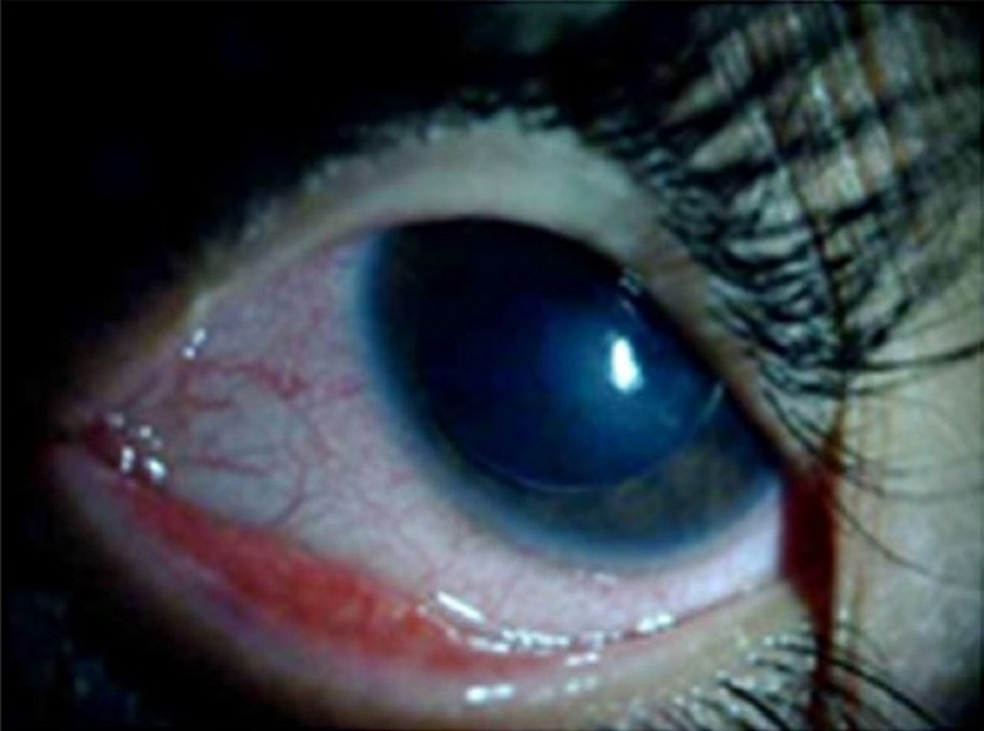
Complete dislocation of the crystalline lens into the anterior chamber accompanied with diffuse corneal stromal edema in the left eye.

Upon ocular examination, testing of visual acuity by Snellen chart was not possible because of the patient’s poor cooperation. The fixation behavior of the right eye appeared normal, but the left eye could fixate without following the target. Due to corneal haziness and complete lens dislocation into the anterior chamber, the determination of the refractive error in this eye was not possible. The previous records indicated the cyclorefraction of the left eye was -12.50/-3.50×90^⸰^. The relative afferent pupillary defect was absent. Slit-lamp examination revealed a dislocation of the crystalline lens into the anterior chamber with diffuse corneal stromal edema in the left eye (Figure 2).

Intraocular pressure (IOP) of the eye was 14 mmHg with an I-care tonometer. In the posterior segment examinations, the media was hazy but the retina was attached. In the fellow eye, the patient had good fixation and could follow moving objects. The cyclorefraction was -8.50/-3.00×90^⸰^ diopter. In this eye, the crystalline lens was mildly subluxated to inferonasal, and the IOP was within a normal range (10 mmHg) and fundus examination was also normal.

**Figure 3:**
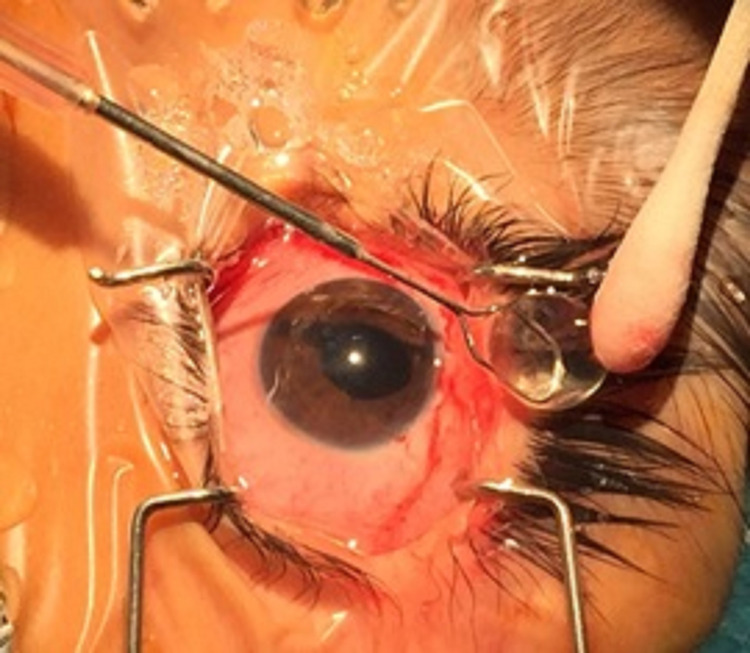
Intraoperative picture of the extracted lens.

Topical eye drops of atropine and betamethasone were started. The patient was also advised to assume a supine position. After 48 hours, due to corneo-lenticular contact and having no response to medications, the patient was prepared for surgery. Due to an increased risk of thromboembolic complications in patients with homocystinuria, the patient underwent general anesthesia after taking anesthetic precautions including proper preoperative hydration and aspirin to prevent deep venous thrombosis. The whole crystalline lens was extracted manually with a lens spoon by making a 5-mm corneoscleral incision superiorly into the anterior chamber after the lens was supported with a viscoelastic agent (Figure 3).

**Figure 4:**
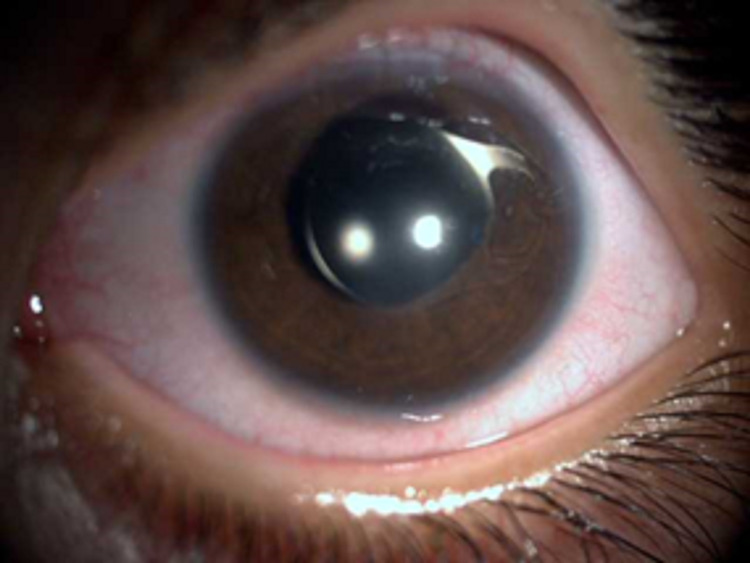
Iris-fixated intraocular lens (IOL) in the left eye.

After lens extraction, no vitreous was present in the anterior chamber. The iris-claw lens was fixated to the anterior iris surface by the enclavation (Figure 4).

The corneoscleral incision was sutured by Nylon 10.0 at the end of the surgery. The patient received topical antibiotics and steroid eye drops for two weeks. One week after surgery, corneal edema was decreased significantly, the fixation became good and he could follow moving objects in the treated eye. Sutures were removed one month after the surgery. At this time, the cyclorefraction improved to -4.00 spherical equivalent. The IOP was 15 mmHg measured by I-care tonometer. No complications such as retinal detachment were identified. At this time, the patient was not compliant with contact lens wear for correcting the refractive error; therefore, the pediatric ophthalmologist prescribed a spectacle to reduce the likelihood of anisometropic amblyopia. Six months after the surgery, iris-fixated intraocular lens (IOL) was in an appropriate location without any corneal endothelial touch. At this time, we scheduled the patient for lens aspiration and implantation of iris-fixated IOL in the right eye, to improve binocular vision and prevent any future possible complications like a scenario of the left eye.

## Discussion

The most frequent cause of homocystinuria is cystathionine β-synthase deficiency, presented as high serum homocysteine level and positive urine homocysteine. Enzyme-deficiency detection in fibroblasts is definitive [2].

The disease involves the central nervous system, eye, skeletal, and vascular system. Progressive mental retardation, skeletal disorders such as chest abnormalities, and thromboembolic episodes are common in these patients [2,4,5]. They need a low methionine diet with pyridoxine (vitamin B6) supplements [6]. Some investigators suggest a combination of multivitamin supplements including folic acid, B6, and B12 to prevent recurrent thromboembolic complications [7].

Ocular abnormalities of the disorder are ectopia lentis, cataract, high myopia, iridodonesis, and retinal detachment [8]. Ectopia lentis, as the main ophthalmic presentation of homocystinuria, has been detected in patients between the ages of 3 and 28 years. The B6-responsive form of the disease is not progressive with the pyridoxine treatment [9]. Lens dislocation is usually bilateral and inferonasal [3]. In a study by Burke et al., only 1 of 19 cases with ocular complications of homocystinuria had unilateral ectopia lentis [10]. Our patient developed lens dislocation and corneal edema in one eye while the fellow eye had just a mild subluxated crystalline lens. This marked asymmetry between eyes is an unusual ocular complication of homocystinuria.

In homocystinuria, more zonular disruption and subsequently increased mobility of the lens are related to age. Therefore, complete anterior lens dislocation can be seen in older ages [9]. Harrison et al. [11] reported that the mean age of patients with complete anterior lens dislocation was 10.2 years. On contrary, our patient was just 6 years old with no compatible history of zonular fibers’ injury.

An anteriorly dislocated lens could cause an acute glaucoma attack. At first, medical treatments including cycloplegic and IOP-reducing drugs should be started. Then, surgical intervention can be contemplated. In a study by Harrison et al. [11], patients with homocystinuria and anterior lens dislocation were first treated with aqueous suppressant and mydriatic eye drops, but all of them needed surgery. Before any decision for surgery, consulting with pediatricians and anesthesiologists is necessary because general anesthesia in these patients may be associated with an increased risk of thrombosis [12]. The most common surgical procedure in these patients is lensectomy via extracting or aspirating the crystalline lens [13]. In most cases, this can be done without vitreous loss. In young patients, the vitreous is adherent to the crystalline lens, and extraction of the lens may lead to vitreous loss and complications like retinal detachment. The rate of vitreous loss and consequent retinal detachment is low, but retinal detachment may be present as a long-term postoperative complication. [11,14,15].

When compliance of these patients for aphakic refractive corrections (including spectacles or contact lenses) is not enough and the risk of amblyopia is high, implantation of IOL is necessary. Some investigators recommend scleral-fixated IOL implantation. Nevertheless, the risk of suture breakage and subsequent IOL dislocation is noticeable in the future and the patients need long-term follow-up [13]. Other studies suggested the implantation of iris-fixated foldable IOLs in children with anterior lens dislocation. They showed that this surgical procedure leads to suitable anatomical and functional outcomes, without any important complications. Also, in comparison to scleral-fixated IOL implantation surgery, transscleral suture fixation is not necessary [16,17]. Our patient refused to wear a high-myopic spectacle. In addition, there were concerns about the correct contact lens use and compliance with long-term follow-up examinations. Therefore, we preferred to implant iris-fixated IOL and correction of the residual refractive error with a spectacle as a combination therapy to reduce the risk of amblyopia.

## Conclusions

In systemic diseases including homocystinuria that cause zonular disintegrations, lens dislocation is usually bilateral and symmetric. However, in unilateral cases especially in the absence of a history of trauma, seeking systemic diseases is necessary. Complete anterior lens dislocation in these patients is an ophthalmic emergency and the dislocated lens must be removed as soon as possible to prevent subsequent ocular complications.
